# The responsiveness of the uterine fibroid symptom and health-related quality of life questionnaire (UFS-QOL)

**DOI:** 10.1186/1477-7525-6-99

**Published:** 2008-11-12

**Authors:** Gale Harding, Karin S Coyne, Christine L Thompson, James B Spies

**Affiliations:** 1 United BioSource Corporation, 7101 Wisconsin Ave, Suite 600, Bethesda, MD 20814, USA; 2 Georgetown University Dept. of Radiology, Room CG201, Bldg. CC, 3800 Reservoir Rd, NW, Washington, DC 20007-2197, USA

## Abstract

**Background:**

A number of noninvasive alternatives to hysterectomy have become available as treatments for uterine fibroids. These alternative therapies, however, may not relieve all symptoms. Consequently, the need for patient-reported outcomes to assess symptom reduction of uterine fibroids has become increasingly important to evaluate the clinical success of patients who choose these alternative therapies. The purpose of the study was to examine the responsiveness of the Uterine Fibroid Symptom and Health-Related Quality of Life Questionnaire (UFS-QOL) with treatment of uterine fibroids.

**Methods:**

The responsiveness of the UFS-QOL was assessed as a post-hoc analysis of patients treated with MRI-guided focused ultrasound thermal ablation (MRgFUS) for uterine fibroids. The UFS-QOL and SF-36 were completed at baseline and months 1, 3, and 6. Patient perceived overall treatment effect (OTE) was assessed at month 3, while satisfaction with treatment was collected at month 6. The responsiveness of the UFS-QOL was examined using effect sizes and change scores by patient-reported overall treatment effect and satisfaction.

**Results:**

A total of 102 women with complete UFS-QOL data were included in the analysis; the mean age was 45 years and 79% were Caucasian. From baseline to 6 months, significant improvements were observed in UFS-QOL Symptom Severity and all Health-Related Quality of Life (HRQL) subscale scores (p < 0.0001). When examining change in general health status over the 6-month follow-up period, significant improvements were noted in all 8 SF-36 subscales. The UFS-QOL was highly responsive with subscale effect sizes ranging from 0.74 for Sexual Function to -1.9 for Symptom Severity. Improvements in UFS-QOL subscales were associated with patient perceptions of perceived benefit and treatment satisfaction.

**Conclusion:**

The UFS-QOL is responsive to treatment for uterine fibroids and is a useful outcome measure for uterine-sparing uterine fibroid treatments.

## Background

Uterine leiomyoma, or uterine fibroids (UF), are the most common neoplasms of the female pelvis, occurring in 20–25% of women of reproductive age [1]. Although benign, fibroid symptoms include pain, bleeding symptoms of menorrhagia and metrorrhagia, bulk symptoms of pressure, heaviness or discomfort in the pelvic area, back, flank, or leg, and urinary frequency. It is the existence and level of bother of uterine fibroid symptoms that lead women to seek treatment, with the current standard of care being abdominal hysterectomy. While a hysterectomy relieves all UF symptoms by removing the uterus, many women are opposed to having a hysterectomy due, in large part, to the undesirable comorbidities such as in-patient hospitalization, prolonged fever, transfusion, scarring, relatively long recovery time to pre-surgical levels of activities [2-4], and elimination of future pregnancies.

A variety of minimally and noninvasive alternatives to hysterectomy that leave the uterus otherwise intact have been introduced as treatments for uterine fibroids [5-9]. These alternative therapies, however, may not relieve all symptoms of UF. Consequently, their use has generated the need for patient-reported outcomes to assess symptom reduction of UF and have become increasingly important to evaluate the clinical success of patients who choose noninvasive treatment of fibroids.

The Uterine Fibroid Symptom and Health Related Quality of Life Questionnaire (UFS-QOL) is a uterine fibroid-specific questionnaire developed to evaluate the symptoms of uterine fibroids and their impact on HRQL. The UFS-QOL has been used in a number of studies of uterine fibroid treatment, including studies of uterine artery embolization [10-15], radiofrequency thermal ablation [16-18], magnetic-resonance-guided ultrasound surgery [19-21] and treatment with medication [22]. This instrument has demonstrated reliability and validity [23] among women with uterine fibroids. However the responsiveness, or the ability of an instrument to accurately detect true change in patient health status [24,25] of the UFS-QOL has not yet been established. To be a useful outcome measure, an instrument must demonstrate responsiveness to treatment effects. The purpose of this study was to evaluate the responsiveness of the UFS-QOL in a clinical study of treatment for uterine fibroids.

## Methods

A post-hoc analysis of data from a prospective, nonrandomized, multi-center study to evaluate the safety and efficacy of MRI-guided focused ultrasound (MRgFUS) thermal ablation to treat uterine fibroids was performed to examine the responsiveness of the UFS-QOL. Inclusion criteria included women who: 1) were pre- or perimenopausal; 2) 18 years of age and older; 3) had completed their family; and 4) presented with symptomatic uterine fibroids, with a score of 41 or greater on the UFS-QOL Symptom Severity screener [26]. Women with uteri > 20-week gestational size or a dominant myoma > 10 cm in diameter were excluded, as were women with a hemotocrit level of < 25%, a major medical disease, or contraindication to MRI (such as a pacemaker). All patients were followed for 6 months to evaluate the change in their symptoms and HRQL. Follow-up visits were completed at 1 week and 1, 3, and 6 months post-treatment. A quiet, private location was provided for patients to complete questionnaires; clinic staff were available to answer any questions. All patients gave written informed consent and the study was approved by an Ethical Committee or Internal Review Board for all centers.

### Measures

#### Uterine Fibroid Symptom and Health Related Quality of Life Questionnaire (UFS-QOL)

The UFS-QOL is a disease-specific questionnaire that assesses symptom severity and HRQL in patients with uterine fibroids [23]. It consists of an 8-item symptom severity scale and 29 HRQL items comprising 6 domains: Concern, Activities, Energy/Mood, Control, Self-consciousness, and Sexual Function. All items are scored on a 5-point Likert scale, ranging from "not at all" to "a very great deal" for symptom severity items and "none of the time" to "all of the time" for the HRQL items. Symptom severity and HRQL subscale scores are summed and transformed into a 0–100 point scale. The Symptom Severity scale and HRQL subscale scores are inversely related with higher Symptom Severity scores indicating greater symptoms while higher HRQL subscale scores indicate better HRQL.

#### Medical Outcomes Study Short-Form 36 (SF-36)

The SF-36 is a generic measure of HRQL and is comprised of 8 subscales: Physical Functioning, Role-Physical, Bodily Pain, General Health, Vitality, Social Functioning, Role-Emotional, and Mental Health [27]. Scores range from 0–100, with higher score indicating better health-related quality of life. SF-36 subscales Social Functioning, Bodily Pain, and Vitality have been shown to be responsive to hysterectomy [28,29].

#### Overall Treatment Effect Scale (OTE)

Patient-rated change was assessed using the OTE Scale [25]. Patients were asked at their 3-month follow-up visit to indicate whether their uterine fibroid symptoms had improved, remained the same, or worsened since the last evaluation. If participants indicated that their symptoms have improved, they were asked to rate the degree of improvement on a 7-point scale from "almost the same, hardly better at all" (1) to "a very great deal better" (7). If subjects indicated that their symptoms worsened, they were asked to rate the degree of worsening on a 7-point scale from "almost the same, hardly worse at all" (-1) to "A very great deal worse" (-7).

#### Treatment Satisfaction

Patients were asked three questions regarding their satisfaction with treatment at month 6. The first item asked patients how satisfied they were with their treatment, with responses based on a 6-point Likert scale, ranging from "very satisfied" to "very dissatisfied." A second item asked patients whether they would recommend their UF treatment to a friend with the same health problems. A four-point response scale was used: "definitely yes," "probably yes," "probably not," and "definitely not." The third item asked patients how effective their treatment was in eliminating their symptoms, with response options based on a 6-point Likert scale, ranging from "very effective, relieved all of my symptoms" to "very ineffective, did not relieve or lessen my symptoms."

### Statistical Analysis

This post-hoc analysis is based on data from patients who were included in the intent-to-treat (ITT) cohort, defined as patients who completed the UFS-QOL at baseline and at least one follow-up assessment. For this responsiveness analysis, missing data was not imputed. Change scores for the UFS-QOL were calculated from baseline to month 3 and baseline to month 6, with the primary analysis based on the change from baseline to month 6. All statistical tests were two-tailed and were conducted with Type I error probability of 0.05. Categorical variables are presented in terms of frequencies and percents; means and standard deviations were calculated for continuous variables.

The responsiveness of the UFS-QOL was examined using patient-reported overall treatment effect at the 3-month assessment. For this analysis, patient responses were examined two different ways. Patients were first grouped based on their rating of overall treatment effect using categorical data responses (i.e., worsened, remained the same, improved). Patient responses were also examined with continuous data responses, whereby improvement is represented by a patient rating greater than or equal to 2; unchanged is represented by a patient rating of -1, 0, and 1; and deterioration is based on a rating of less than or equal to -2. Due to sample size issues, UFS-QOL change scores were compared among the "worse" or "same" versus "improved" patient groups using general linear models (PROC GLM) controlling for age and baseline UFS-QOL scores.

UFS-QOL change scores by patient-reported satisfaction were also examined. Due to sample size issues, the satisfaction items were dichotomized according to whether the patients were satisfied with treatment (satisfied/not satisfied), whether they would recommend the treatment to a friend (yes/no), and whether the treatment was effective in eliminating symptoms (effective/not effective). UFS-QOL change scores were compared among the above-noted patient groups using PROC GLM controlling for age and baseline UFS-QOL scores.

Effect sizes, a quantitative measure of change that standardizes the comparison between groups, were calculated for each UFS-QOL and SF-36 subscale by using the difference in mean scores from baseline to month 6 and dividing by the standard deviation of baseline scores of all participants. Effect size was interpreted as small (0.20), moderate (0.50), or large (0.80) according to the guidelines proposed by Cohen [30]. All analyses were performed using SAS^® ^Version 9.1.3.

## Results

Of the 108 patients who received UF treatment and completed a baseline UFS-QOL, 5 patients did not complete a 6-month follow-up assessment and 1 did not provide a baseline UFS-QOL assessment, thus resulting in a sample of 102 patients. Among the study participants, the mean age was 45 years, 79% (n = 81) were Caucasian, with a mean body mass index (BMI) of 26.0 (Table 1).

**Table 1:** Baseline demographic and clinical characteristics

**Characteristic**	**Total N = 102**
**Age **mean years (SD)	45.0 (4.8)

**Race**	

Caucasian	81 (79.4%)

African-American	11 (10.8%)

Asian	3 (2.9%)

Hispanic	1 (1.0%)

Other	6 (5.9%)

**BMI **mean (SD)	26.0 (5.4)

**History of other gynecological disease **(n; % yes)	9 (8.8%)

The mean change in UFS-QOL subscale scores from baseline to 6 months indicates substantial patient improvement, with the Symptom Severity and all HRQL subscales demonstrating statistically significant changes (p < 0.0001) (Table 2). Mean change scores were all greater than or equal to 20 points, ranging from 20.2 for Concern to -27.8 for Symptom Severity. In addition to Symptom Severity, the HRQL subscales of Activities and Self-consciousness demonstrated the most change in patient-perceived health status, with mean changes scores of 26.1, and 25.7, respectively. The majority of improvement occurred within the first 3 months after treatment (mean change scores ranging 19.7 for Sexual Function to 25.7 for Self-consciousness) with continuing, but slight, improvements to 6 months. All of the UFS-QOL subscales demonstrated large effect sizes from baseline to month 6, ranging from 0.7 for Sexual Function to -1.9 for Symptom Severity (Table 2). From baseline to month 3, the effect sizes were of similar magnitude, ranging from 0.7 for Sexual Functioning to -1.7 for Symptom Severity.

**Table 2:** UFS-QOL scores at baseline, 3, and 6 months

**UFS-QOL Subscales**	**Baseline N = 102 Mean (SD)**	**Month 3^1 ^N = 100 Mean (SD)**	**Mean Change Score^2^**	**Month 6 N = 102 Mean (SD)**	**Mean Change Score^2^**	**6 month Effect Size**
**Symptom Severity**	61.5 (14.7)	37.1 (20.2)	-24.4	33.9 (19.0)	-27.8	-1.87

**Concern**	45.8 (26.2)	66.0 (23.9)	20.2	65.6 (24.9)	20.2	0.77

**Activities**	46.9 (21.4)	70.0 (23.4)	23.1	72.7 (23.1)	26.1	1.20

**Energy/mood**	48.8 (22.8)	71.6 (21.9)	22.8	72.2 (21.6)	23.8	1.04

**Control**	48.5 (24.3)	71.5 (25.2)	23.1	72.9 (24.5)	24.6	1.01

**Self-consciousness**	39.3 (26.8)	65.0 (28.3)	25.7	64.4 (27.4)	25.7	0.95

**Sexual Function**	50.8 (28.7)	70.5 (27.3)	19.7	71.7 (29.0)	21.2	0.74

**Total HRQL Score**	46.8 (18.3)	69.6 (20.6)	22.8	70.5 (20.6)	24.0	1.30

^1 ^Month 3 UFS-QOL, n = 100 due to missing data

^2 ^All change score P values < 0.0001

Similar patterns of patient perceived improvement were found with the SF-36, although the magnitude of change was less than that observed with the UFS-QOL (Table 3). Mean change scores from baseline to month 6 were all statistically significant, suggesting improved HRQL, and ranged from 5.5 (p = 0.007) for General Health to 25.2 (p < 0.0001) for Role-Physical. As was the case with the UFS-QOL, the majority of patient-perceived improvement occurred within 3 months after treatment, with sustained improvement at 6 months. Effect sizes for the SF-36 were of a lesser magnitude than those compared to the UFS-QOL, ranging from 0.3 for General Health to 0.9 for Bodily Pain and Vitality at month 6 (Table 3).

**Table 3:** SF-36 scores at baseline, 3, and 6 months

**SF-36**	**Baseline** **N = 102** **Mean (SD)**	**Month 3**^1,2^ **N = 101** **Mean (SD)**	**Mean Change Score**	**Month 6**^2^ **N = 102** ** Mean (SD)**	**Mean Change Score**	**6 month Effect Size**
**Physical Function**	72.4 (24.3)	85.0 (19.6)	12.6	85.0 (19.3)	12.8	0.54

**Role-Physical**	45.3 (41.8)	68.9 (39.5)	23.6	70.1 (40.0)	25.2	0.62

**Body Pain**	52.4 (22.2)	69.3 (23.0)	17.0	71.1 (24.1)	19.0	0.87

**General Health**	65.9 (19.9)	72.1 (20.9)	6.2	71.3 (21.0)	5.5	0.29

**Vitality**	40.9 (21.0)	59.4 (20.7)	18.6	59.5 (20.2)	18.9	0.92

**Social Functioning**	60.8 (27.5)	79.1 (22.1)	18.4	80.1 (21.7)	19.7	0.73

**Role-Emotional**	57.7 (40.2)	81.0 (34.2)	23.3	77.6 (34.1)	20.4	0.52

**Mental Health**	62.9 (17.1)	74.4 (15.6)	11.5	74.3 (14.0)	11.6	0.69

^1 ^Month 3 SF-36, n = 101 due to missing data

^2 ^All change score P values < 0.001

When using the Overall Treatment Effect scale responses to examine the UFS-QOL's responsiveness, the UFS-QOL demonstrated significantly greater change in patients who reported that their fibroid symptoms "improved" (61.6%) at 3 months compared to those who reported that their symptoms were the "same" or "worse" (38.4%). The Symptom Severity subscale and all of the HRQL subscales demonstrated significantly greater change for those who reported improvement (OTE score > 2) compared to those who did not (OTE score ≤ 1). Change scores for "improved" patients ranged from 24.8 to 31.5 while change scores for patients who were the "same" or "worse" ranged from 10.1 to 17.5. The mean difference in change scores between the "improved" and "same/worse" groups ranging from 9.0 for Activities to 15.2 for Self-consciousness (Table 4).

**Table 4:** Change in UFS-QOL scores from baseline to month 3, by overall treatment effects

**UFS-QOL Subscales LS Mean (SE)**	**Worse/Same (OTE ≤ 1) N = 38**	**Improved (OTE ≥ 2) N = 61**	**Difference Between Improved – Same/Worse**	**P Values**^2^
**Symptom Severity**	-16.8 (3.0)	-29.6 (2.3)	-12.8	0.001

**Concern**	12.3 (3.3)	24.9 (2.6)	12.6	0.004

**Activities**	17.5 (3.2)	26.5 (2.5)	9.0	0.03

**Energy/mood**	15.6 (2.9)	27.4 (2.3)	11.8	0.002

**Control**	15.6 (3.2)	27.8 (2.6)	12.2	0.004

**Self-consciousness**	16.3 (3.7)	31.5 (2.9)	15.2	0.002

**Sexual Function**	10.1 (3.9)	24.8 (3.1)	14.7	0.004

**Total HRQL Score**	15.6 (2.8)	27.1 (2.1)	11.5	0.001

^1 ^PROC GLM model controlling for age and baseline UFS-QOL Score of patient was performed. P values are: * < 0.05., ** < 0.01, *** < 0.001.

^2 ^T-tests between means for "worse/same" versus "improved" patients were performed.

Significant differences were present among all UFS-QOL scales scores when comparing satisfied and dissatisfied patients (Table 5). With respect to satisfaction with treatment, patients who were satisfied demonstrated significantly greater improvements in the Symptom Severity and all UFS-QOL HRQL subscales compared to those who were dissatisfied (all p < 0.02). The difference in change was the highest for the subscales of Concern (mean difference = 20.0), Activities (mean difference = 20.9), Sexual Function (mean difference = 22.2), and Control (mean difference = 22.5). Similar results were found among patients who reported that their treatment was effective compared to those who reported that their treatment was not effective, with all subscales indicating significantly greater improvement among those who reported that their treatment was effective (all p < 0.001) (Table 5). No statistically significant differences in UFS-QOL change scores were observed between those who indicated that they would recommend treatment to a friend compared to those who would not.

**Table 5:** Change in UFS-QOL scores from baseline to 6 months, by patient satisfaction

**UFS-QOL Subscales**^1^ **LS Mean (SE)**	**Satisfaction with Treatment**			**Effectiveness in Eliminating Symptoms**		
	
	**Satisfied N = 83**	**Dissatisfied N = 15**	**P Values**	**Effective N = 78**	**Ineffective N = 20**	**P Values**
**Symptom Severity**	-29.4 (1.9)	-14.7 (4.6)	0.004	-30.8 (2.0)	-13.9 (3.9)	0.0002

**Concern**	23.1 (2.4)	3.1 (5.7)	0.002	24.2 (2.4)	2.8 (4.8)	0.0002

**Activities**	29.3 (1.9)	8.4 (4.6)	< .0001	30.0 (2.0)	8.9 (3.9)	< .0001

**Energy/mood**	26.7 (1.9)	9.7 (4.4)	0.0006	27.9 (1.9)	8.7 (3.7)	< .0001

**Control**	28.2 (2.0)	5.7 (4.8)	< .0001	28.8 (2.1)	8.2 (4.1)	< .0001

**Self-consciousness**	27.8 (2.3)	13.3 (5.4)	0.02	29.7 (2.3)	11.6 (4.6)	0.0008

**Sexual Function**	24.8 (2.9)	2.6 (6.9)	0.004	27.3 (2.9)	-0.2 (5.7)	< .0001

**Total HRQL Score**	27.1 (1.8)	7.7 (4.1)	< .0001	28.2 (1.8)	7.7 (3.5)	< .0001

^1 ^Controlling for age and baseline UFS-QOL score. Scheffe's post-hoc comparisons were performed.

## Discussion

The UFS-QOL demonstrated responsiveness to change among women treated with MRgFUS, suggesting that the instrument is sensitive to change in patient-perceived Symptom Severity and HRQL. The UFS-QOL was able to detect patient improvement from baseline to 3 months and baseline to 6 months, with mean change scores greater than or equal to 20 points for all subscales. The large change scores are consistent with the large effect sizes observed for the Symptom Severity and HRQL subscales, all of which were 0.80 or higher at six months with the exception of Sexual Function, which was 0.70. Using Cohen's guide for social phenomena [30], these values can be interpreted as large for Symptom Severity, Concern, Activities, Energy/Mood, Control, Self-consciousness, and total HRQL, and medium for Sexual Function. Our findings are consistent with those recently reported by the Fibroid Registry Outcomes for Outcomes Data (FIBROID), a registry study of over 2,000 patients undergoing uterine embolization for leiomyomata [15]. Six-month follow-up data from this study also reported large change scores of more than 35 points from baseline for both Symptom Severity and Total HRQL.

When using ancillary measures as benchmarks for patient improvement, the UFS-QOL was able to discriminate among levels of change in patient-reported overall treatment effect and satisfaction. With respect to overall treatment effect, the UFS-QOL was able to demonstrate significantly greater improvements in Symptom Severity and HRQL among those who reported that their fibroid symptoms improved compared to those who reported no improvement. The UFS-QOL was also able to discriminate between patients who were satisfied with treatment (compared to those who were not) and between patients who reported that their treatment was effective (compared to those who did not) in all subscales of HRQL. The UFS-QOL was not able to discriminate between patients who indicated that they would recommend the treatment to a friend compared to patients who would not, but this may be an indication that this particular item is not a good measure of patient satisfaction.

In prior research, the SF-36 subscales Social Function, Bodily Pain, and Vitality have been shown to be responsive to hysterectomy [28,29]. In this study, effect sizes for these subscales ranged were 0.7, 0.9, and 0.9 for Social Functioning, Bodily Pain, and Vitality respectively, indicating moderate to large effect sizes. In addition, all SF-36 subscales with the exception of General Health were able to detect changes in patient perceived health status over time. While the SF-36 was able to demonstrate adequate responsiveness to change in the health status of patients treated for uterine fibroids, our findings suggest that the disease-specific UFS-QOL with its focus solely on UF symptoms was clearly more responsive with change score differences and effect sizes of a much greater magnitude compared to the SF-36.

The approach we used to assess the responsiveness of the UFS-QOL is consistent with that outlined by Juniper et al. [24] by addressing the following questions:

1. In patients who truly change their health status, can we measure this change by comparing baseline and follow-up scores?

2. Is the instrument able to distinguish between patients whose health status changes and those who remain stable?

3. What is the magnitude of the instrument's responsiveness?

A major strength of this study is the demonstrated responsiveness of the UFS-QOL in several different analyses. Greater confidence can be placed in the interpretation of clinically significant effects with consistent evidence from multiple sources. In the current study, the UFS-QOL Symptom Severity and HRQL subscale scores between consecutive assessments demonstrated improved symptoms and HRQL for within-patient comparisons. Discriminant ability with patient perceptions was also observed in addition to the large effect sizes. The consistency of our findings strongly suggests that the observed score changes are valid and clinically meaningful.

Due to ethical considerations, a placebo group (or sham treatment) could not be included in this study. As such, a limitation of this study is that change in the UFS-QOL due to placebo effect cannot entirely be ruled out, although follow-up of this patient cohort shows that the reductions in symptom severity appear to be maintained over time. Future research examining the responsiveness of the UFS-QOL with placebo-controlled alternative therapies for which the uterus remains in tact is needed to address this limitation.

Patient-reported outcomes of symptom severity and HRQL are becoming increasingly important for evaluating treatment of uterine fibroids within the clinical setting. With the increasing availability of noninvasive therapies to hysterectomy, it will be especially important to assess symptom reduction of uterine fibroids and evaluate the clinical success of patients who choose these treatment options. Psychometrically sound patient-reported outcome measures, with demonstrated reliability, validity, and responsiveness, are needed to evaluate interventions among this patient population. The current study indicates that the UFS-QOL is responsive to change in patient health status and able to discriminate between those who improve with treatment for uterine fibroids and those who do not. Consequently, the UFS-QOL appears to be a useful evaluative tool for assessing symptoms and HRQL in studies among patients with uterine fibroids.

## Conclusion

The UFS-QOL is responsive to treatment for uterine fibroids and is a useful outcome measure for uterine-sparing uterine fibroid treatments.

## Abbreviations

FIBROID: Fibroid Registry Outcomes for Outcomes Data; HRQL: Health-Related Quality of Life; ITT: Intent-to-Treat; MRgFUS: MRI-guided Focused Ultrasound thermal ablation; OTE: Overall Treatment Effect; PROC GLM:General Linear Models comparison; SF-36: Short-Form 36; UF: Uterine Fibroids, also known as uterine leiomyoma; UFS-QOL: Uterine Fibroid Symptom and Health-Related Quality of Life Questionnaire.

## Competing interests

Funding for this research was provided by InSightec-TxSonics, Inc.

## Authors' contributions

All coauthors contributed equally to this manuscript. All authors read and approved the final manuscript.
